# The Response of Durum Wheat to the Preceding Crop in a Mediterranean Environment

**DOI:** 10.1155/2014/717562

**Published:** 2014-10-21

**Authors:** Laura Ercoli, Alessandro Masoni, Silvia Pampana, Marco Mariotti, Iduna Arduini

**Affiliations:** ^1^Scuola Superiore Sant'Anna, Piazza Martiri della Libertà 33, 56127 Pisa, Italy; ^2^Department of Agriculture, Food and Environment, University of Pisa, Via del Borghetto 80, 56124 Pisa, Italy; ^3^Department of Veterinary Science, University of Pisa, Via delle Piagge 2, 56124 Pisa, Italy

## Abstract

Crop sequence is an important management practice that may affect durum wheat (*Triticum durum* Desf.) production. Field research was conducted in 2007-2008 and 2008-2009 seasons in a rain-fed cold Mediterranean environment to examine the impact of the preceding crops alfalfa (*Medicago sativa* L.), maize (*Zea mays* L.), sunflower (*Helianthus annuus* L.), and bread wheat (*Triticum aestivum* L.) on yield and N uptake of four durum wheat varieties. The response of grain yield of durum wheat to the preceding crop was high in 2007-2008 and was absent in the 2008-2009 season, because of the heavy rainfall that negatively impacted establishment, vegetative growth, and grain yield of durum wheat due to waterlogging. In the first season, durum wheat grain yield was highest following alfalfa, and was 33% lower following wheat. The yield increase of durum wheat following alfalfa was mainly due to an increased number of spikes per unit area and number of kernels per spike, while the yield decrease following wheat was mainly due to a reduction of spike number per unit area. Variety growth habit and performance did not affect the response to preceding crop and varieties ranked in the order Levante > Saragolla = Svevo > Normanno.

## 1. Introduction

Durum wheat (*Triticum durum* Desf.) is a traditional crop included in most dishes and food products consumed in the Mediterranean basin. Durum wheat producers are currently under pressure to maintain profitability against a background of environmental constraints, high fertiliser costs, and increasing quality demands of the pasta industry. Therefore, the development of cropping strategies that reduce input costs and increase the efficiency of fertilisers and simultaneously reduce the risks of nitrate leaching and denitrification is crucial [1].

A considerable volume of the literature addresses the effects of preceding crop on wheat. Kirkegaard et al. [2] carried out a survey of the literature on the effects of break crops, that is, crops interrupting the sequence of continuous wheat, and showed mean yield benefits of up to 20% or more, the magnitude of response depending on site, weather conditions, and other aspects of crop management. Overall, the inclusion of N_2_-fixing plants in crop rotations provided positive crop sequential enhancements to the yields of other crops compared to the nonleguminous crops [3]. Nix et al. [4] estimated the yield reduction in continuous wheat: the second successive wheat crop yields approximately 10% lower than the first, and the third wheat crop can yield 10–15% below the second. Lower yields for wheat planted after a high-residue crop with a high C/N ratio compared with wheat planted after legumes have been attributed to greater immobilisation of N [5–7] or reduced disease control [8, 9]. Autotoxicity may also result from the presence of phytotoxins produced by the preceding wheat crop building up in the soil with continuous culture over time [3, 10]. Conversely, long-term studies in Mediterranean environments have produced evidence that, with adequate annual fertilisation and effective weed control, continuous wheat cropping may be used for many years without significant yield decline [11, 12].

Despite the many studies, a comprehensive picture of grain development under different environmental conditions has yet to emerge, particularly for profitable crop management. The objective of this study was to examine the impact of the preceding crops alfalfa, maize, sunflower, and wheat on the yield of durum wheat in a rain-fed cold Mediterranean environment. Four varieties were included in research over two seasons to test the hypothesis that variety growth habit and performance would affect the response to the preceding crop.

## 2. Materials and Methods

### 2.1. Experimental Conditions

Trials were carried out during two consecutive growing seasons (2007-2008 and 2008-2009) in Pisa, Italy (43°40′N, 10°19′E, 1 m a.s.l.). Seawater intrusion is not of concern in the area, as soil chemical analyses never indicated salinization in the 0–90 cm soil layer. The climate at the experimental site is characterised as cold humid Mediterranean with 120-year average of mean annual maximum and minimum daily air temperatures of 20.2 and 9.5°C, respectively, and annual precipitation of 971 mm, with 688 mm received during the period of durum wheat cultivation, which is from November to July [14]. The month normally receiving the greatest amount of rainfall is December (13% of total).

Soil samples (0–40 cm depth) were collected in November 2007 and December 2008 before wheat seeding. Samples were thoroughly mixed, air-dried, and ground and after sieving, material <2 mm was analysed. Particle-size distribution was determined by the hydrometer method [15]. The pH was measured in a 1 : 1 soil-H_2_O suspension [16]. A modified wet-digestion Walkley-Black method [17] was used for the organic matter determination. Total soil N was measured by the modified Kjeldahl method [18]. Nitrate N was determined by the ion-selective electrode method [19], available P by the Olsen method, [20] and available K by the Dirks-Sheffer method [21]. Principal soil physical and chemical properties were 37% sand (2 mm > *∅* > 0.05 mm); 39% silt (0.05 m > *∅* > 0.002 mm); 24% clay (*∅* < 0.002 mm); 7.0 pH; 1.6% organic matter; 0.8 g kg^−1^ total nitrogen; 6.0 mg kg^−1^ available P; and 149 mg kg^−1^ available K. Soil texture was loam and soil type was Typic Xerofluvents according to the USDA soil taxonomy.

The durum wheat varieties Levante, Normanno, Saragolla, and Svevo (Breeder: Produttori Sementi Spa, Bologna, Italy) were established following alfalfa, maize, sunflower, and durum wheat on 25 November 2007 and 16 December 2008. Medium to high yielding varieties of durum wheat were chosen representing the high genotypic variability within commercial varieties in growth cycle length and grain quality. Levante, Normanno, Saragolla, and Svevo are new durum wheat varieties (released, resp., in 2002, 2002, 2004, and 1996) that are widely cultivated in Central Italy and differ in cycle length and grain quality. Levante and Normanno are medium maturing, Saragolla is early maturing, and Svevo is very early maturing. All have a high yellow index, and Levante and Svevo have a very high protein concentration, while Normanno is high and Saragolla medium-high. Finally, gluten quality is high in Normanno and Saragolla, medium in Levante, and low in Svevo.

Sixteen treatments, consisting of four preceding crops (alfalfa,* Medicago sativa* L., maize,* Zea mays* L., sunflower,* Helianthus annuus* L., and bread wheat,* Triticum aestivum* L.) and four varieties, were compared in both years. In Central Italy, wheat is typically rotated with maize and sunflower and to a lesser extent with alfalfa. Preceding crops were grown in adjacent fields and were rain-fed. The trial was laid out in a strip-plot design, with preceding crop as the main treatment with three replicates. Varieties were randomised within the main treatment. The area of each plot was 250 m^2^. The preceding crops wheat, sunflower, and maize were established and harvested in the years before the experiment, while alfalfa was established three years previously and terminated in the preceding year. Maize, sunflower, and wheat were grown for grain and alfalfa for hay. Following conventional cultural practices, alfalfa was cut four times per year. The biomass left in the field was approximately 500 g m^−2^ dry weight for alfalfa, 600 g m^−2^ for sunflower, 700 g m^−2^ for maize, and 400 g m^−2^ for wheat. Crops were cultivated following the technique normally applied in the area. Wheat was harvested in early July, maize was in mid-September, sunflower was in early August, and alfalfa was in early October.

Soil tillage before durum wheat sowing was performed by subsequent ploughing, disking (16 October 2007 and 5 October 2008), and harrowing (16 November 2007 and 15 December 2008). The previous crop residuals were incorporated into the soil by ploughing. Phosphorus and potassium were applied immediately before soil tillage as triple mineral phosphate (Ca(H_2_PO_4_)_2_ *·*H_2_O) and potassium sulphate (K_2_SO_4_), at rates of 35 kg ha^−1^ P and 66 kg ha^−1^ K. Nitrogen was applied at 150 kg N ha^−1^, split into three doses of 30, 60, and 60 kg N ha^−1^, the first before soil harrowing as ammonium sulphate and the others as urea at pseudostem erection (24 March 2008 and 24 March 2009) and at first node detectable (7 April 2008 and 8 April 2009). Growth stages of pseudostem erection and first node detectable were individuated following the scale of Zadoks [13], corresponding, respectively, to GS30 and GS31. The fertilisers N, P, and K rates were calculated based on the balance approach. Durum wheat varieties were sown in rows 15 cm apart at the rate of 400 viable seeds m^−2^. Weed control was performed with a preemergence application of trifluralin.

### 2.2. Data Collection and Analyses

For all treatments, timings of the stage of 1st leaf emergence (GS10), 5th leaf unfolded (GS15), pseudostem erection (GS30), anthesis (GS60), and physiological ripening (GS90) were recorded, expressing in thermal time the duration of the periods between stages (Table 1). Thermal time was calculated following McMaster and Wilhelm [22], assuming 2°C as the base temperature [23]. At GS90 (22 June 2008 and 30 June 2009) wheat plants from a 1 m^2^ area were manually cut at ground level and were partitioned into stems (culms), leaves, nonseed portion of inflorescences (chaff), and caryopses (grain). For dry weight determination, samples from all plant parts were oven dried at 65°C up to constant weight. The number of spikes per unit area, mean kernel weight, number of spikelets per spike, and number of kernels per spike were measured at maturity. Harvest index (HI) was calculated as (dry weight of grain/dry weight of aerial plant part) × 100. Samples of each plant part were analysed for nitrogen concentration (modified Kjeldahl method, [18]); N contents were calculated by multiplying N concentration by dry weight. Since the effects of treatments were similar on leaves, culms, and chaff, data were combined together and hereafter referred to as the vegetative plant part. Spike fertility index (SFI) was calculated following the method of Abbate et al. [24] as the quotient between grain number/m^2^ and spike chaff dry weight/m^2^ at maturity. The durum wheat plots were evaluated for disease incidence and severity by visual assessment of symptoms of fungal infection.

### 2.3. Statistical Analysis

Results were treated with ANOVA. The main effects of year, preceding crop, variety, and their interactions were tested for all measured characters. The CoHort software package version 6.4 (CoHort software, Monterey, CA, USA) was used. Significantly different means were separated at the 0.05 probability level by the least significant difference test [25].

## 3. Results

### 3.1. Weather Conditions

Rainfall varied yearly over the 2-year period: in 2007-2008 it was similar to the 120-year average in the area and was by 29% higher in 2008-2009 (Table 1). In 2007-2008, rainfall was well distributed through the wheat cycle and was more favourable for wheat growth and development. In 2008-2009, high rainfall occurred during autumn and low rainfall during spring. The variable rainfall patterns experienced during the study are typical for this wheat-producing area of the Mediterranean basin. Temperature was similar to the long-term average in both seasons, as the thermal time up to maturity was 2574°C in the long-term average versus 2642°C d in 2007-2008 and 2623°C d in 2008-2009 (Table 1). Accumulated growing degree days during wheat development phases were also alike in the two years.

### 3.2. Preplanting Nitrate Content in Soil

The soil NO_3_-N content before wheat sowing in both years was affected by the preceding crop. In the autumn of 2007 and 2008, more residual soil nitrate was available after alfalfa and values decreased in the following order: alfalfa, maize, sunflower, and wheat. Lower values were recorded in autumn 2008 after all crops (Table 2). Compared to the case of wheat as the preceding crop, soil NO_3_-N left after alfalfa was about threefold, resulting in an average of approximately 55 kg N ha^−1^ in the 0–40 cm profile.

### 3.3. Grain Yield and Yield Components

Durum wheat grain yield and yield components were influenced by year, variety, and previous crop and by various interactions. The preceding crop differently affected grain yield according to the year of cultivation: in 2007-2008 grain yield was highest following alfalfa and, compared to this preceding crop, was 33% lower following wheat. In 2008-2009 no difference in grain yield was detected among preceding crops (Table 3). In 2008-2009, wheat yields were low (less than 400 g m^−2^) due to high rainfall during the vegetative phase of plant development and low rainfall during the reproductive phase, leading to delayed emergence and prolonging the period during which plants are more sensitive to waterlogging. Moreover, poor crop establishment was associated with stunted growth due to high N leaching.

Differences in grain yield between years and preceding crops were due to variations in yield components. Mean kernel weight was 46% higher in 2007-2008 compared to 2008-2009 and was not affected by the preceding crop (Table 4). Conversely, the average number of kernels per spike in 2007-2008 compared to 2008-2009 was 38, 18, and 20% higher following alfalfa, maize, and sunflower, respectively, while wheat as preceding crop did not produce variation between years (Table 3). The number of spikes per unit area was affected by the preceding crop, with the lowest value for durum wheat following wheat compared to the other precessions (Table 4).

Grain yield of varieties was different in the two years (Table 5). In 2007-2008 production ranked in the order Levante > Saragolla = Svevo > Normanno, while in 2008-2009 production of all varieties did not differ. Differences in grain yield between years and varieties were due to variations in the number of kernels per spike and mean kernel weight, as the number of spikes per unit area did not vary (Table 5). Mean kernel weight did not vary among varieties and was higher in 2007-2008 as previously discussed. The number of kernels per spike of Levante, Saragolla, and Svevo was, respectively, 25, 28, and 20% higher in 2007-2008 (Table 5). Grain yield was not affected by the interaction between variety and precession, as varieties responded similarly to the preceding crop (results not shown).

The response of the vegetative growth to the interaction year × preceding crop was similar to the same interaction in grain yield with maize and wheat as the preceding crops; vegetative growth was similar in the two years, while it was increased in 2007-2008 by 73% with alfalfa preceding and by 47% with sunflower preceding (Table 3). Similar to grain, the vegetative plant part of Levante, Saragolla, and Svevo was higher in 2007-2008 (Table 5).

The harvest index was 46% in 2007-2008 and 42% in 2008-2009 and was not modified by the preceding crop or variety (results not shown).

The number of spikelets per spike did not change in the two years but varied according to the variety and the preceding crop (Table 4). Varieties ranked in the following order: Levante > Saragolla = Normanno > Svevo, and alfalfa, maize, and sunflower as preceding crops affected similarly spike size, while wheat as preceding crop reduced by 8% the number of spikelets per spike.

Comparing among the treatments, spike fertility index (SFI) differed between years and precessions. SFI was 20% higher in 2008 and alfalfa precession resulted in a 24% increase of SFI compared to other precessions (Table 4).

### 3.4. Nitrogen Uptake

**Figure 1:**
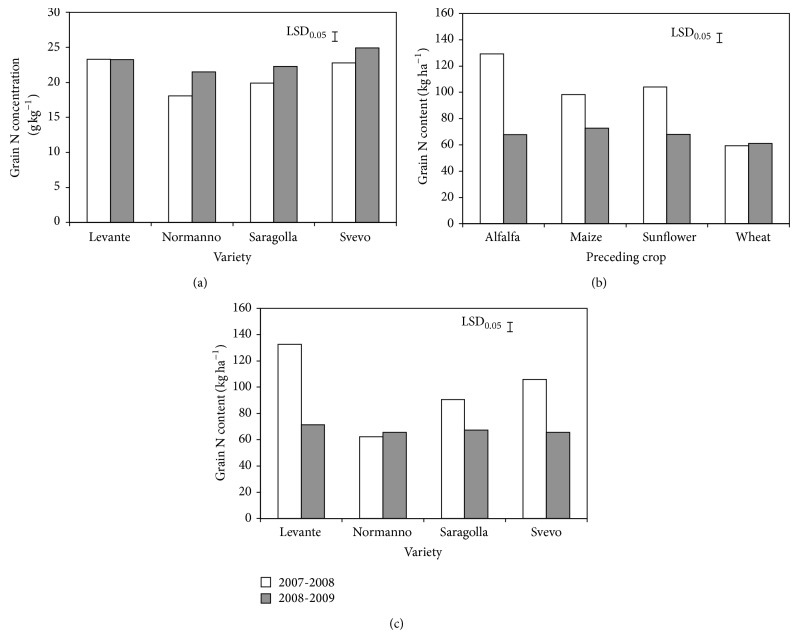
Grain N concentration as affected by variety (a) and grain N content as affected by preceding crop (b) and variety (c) mean effects. Bars indicate Fisher's LSD test at *P* ≤ 0.05.

Grain N concentration was not affected by the preceding crop but responded to the interaction year × variety, increasing by 20, 12, and 10% in 2008-2009 compared to 2007-2008, respectively, in Normanno, Saragolla, and Svevo (Figure 1(a)). Conversely, N concentration of grain of Levante did not change in the two years.

Nitrogen concentration in the vegetative plant part was not affected by the preceding crop and did not vary in the tested varieties but was mainly modified by the year of cultivation. Averaged over preceding crops and varieties, N concentration in the vegetative plant part was 6.6 g kg^−1^ in 2007-2008 and 4.0 g kg^−1^ in 2008-2009.

Following the patterns of dry weight and N concentration, N content of grain was affected by the interactions year × precession and year × variety. The effect of the preceding crop on grain N content was different in the two years: in 2007-2008 grain N content following alfalfa was the highest, that following maize and sunflower was intermediate, and that following wheat was the lowest while in 2008-2009, grain N content was lowest and unaffected by the preceding crop (Figure 1(b)). In 2007-2008, grain N content in Levante, Saragolla, and Svevo was higher, while in 2008-2009 values in the four varieties were similar (Figure 1(c)). The highest N content in grain was recorded in Levante. Finally, grain N content in Normanno was lowest in both years, without any appreciable difference.

Nitrogen content of the vegetative plant part was not modified by the preceding crop or variety but was higher in 2008-2009 than in 2007-2008 (27.8 versus 23.5 kg ha^−1^, data not shown).

## 4. Discussion

An ideal preceding crop for wheat should permit taking advantage of available water and nutrients while disrupting weed and disease cycles, resulting in decreased requirements for fertilisers, pesticides, and herbicides [26]. In our research, the response of durum wheat to the preceding crop was high in the favourable season (2007-2008) but was absent in the unfavourable one (2008-2009). In the most favourable season, durum wheat grain yield was highest following alfalfa and, compared to this preceding crop, was 33% lower following wheat. Yield components are probably linked to increases in the yield potential of wheat following the tested preceding crops. Lower wheat yields in 2008-2009 were due to high rainfall during the vegetative phase of plant development and low rainfall during the reproductive phase, leading to delayed emergence and prolonging the period during which plants are most sensitive to waterlogging. Moreover, poor crop establishment was associated with stunted growth due to high N leaching.

The yield increase of durum wheat following alfalfa was mainly due to an increased number of spikes per unit area and number of kernels per spike, while the yield decrease following wheat was mainly due to a reduction of the number of spikes.

Nitrogen availability during both early and late crop development is a possible cause for the differences in growth and yield of durum wheat following the tested preceding crops. Differential N availability results from the balance between mineralization and immobilization of N in soil organic matter. The amount of released or immobilized N depends on the biomass and the C/N ratio of residues left in the field by the preceding crop. We did not determine the C/N ratio of residues but, according to [2], net N mineralization is expected from alfalfa residues, because of a C/N ratio lower than 30; while net N immobilization is favored from maize, sunflower, and wheat residues, due to a C/N ratio greater than 40. More residual soil nitrate N was available before durum wheat planting following alfalfa [2]. Observation of increased soil nitrate N levels following alfalfa, likely due to the mineralization of N in legume organic matter residues [27], confirms previous findings that net soil N mineralisation following alfalfa was 30 to 40% greater than that following maize or soybean [28]. Results of Gaiser et al. [29] suggest that alfalfa as the preceding crop supports deeper rooting and higher rooting density of following spring wheat, enhancing access to water and nutrients in deeper soil layers.

The assumption of an effect of late N availability is supported by the response of the spike fertility index, which was increased in durum wheat following alfalfa compared to the other precessions. The SFI is a complex trait that includes spike structure, that is, the assimilate partitioning inside the spike; development and survival of florets; and grain set. These processes follow a general pattern that is genetically predetermined but whose speed can be modified by environmental conditions, such as N fertilisation [30, 31]. This experiment showed that durum wheat following alfalfa, while initiating a relatively similar number of spikelets to that of durum wheat after other crops, set more grains per spike. Thus it is likely that the higher N availability in soil owing to the release of N from the mineralisation of legume residues allows higher floret survival at anthesis.

A possible explanation for differences due to preceding crops is also the toxic effect of allelochemicals released by wheat residues, affecting crop establishment [9, 10, 32], which were not measured in the present study. It could be argued also that the durum wheat plants following wheat might take up N at lower rates because of a smaller root system, presumably due to the toxic effect of allelochemicals. Moreover, allelochemicals may also have an indirect effect on plant growth by affecting potentially beneficial rhizosphere microorganisms, for example, by inhibiting mycorrhizal formation or microorganisms involved in nutrient cycling [33].

Weed development was low and no evidence of disease was documented during the wheat growth and no relation was found with preceding crops. In particular,* Fusarium* sp. infection, representing one of the most harmful diseases in our environment, did not occur following either host crops like maize or wheat or nonhost crops like alfalfa or sunflower [34]. Probably, climatic conditions during grain filling in both years were not favorable for infection, as* Fusarium* sp. is favoured by rainy and humid weather during grain filling [35].

Genotypic effects were mainly observed for grain yield and grain number, but variety growth habit and performance did not affect the response to preceding crop, in that no change in the rank order of varieties was observed. The rank order of the varieties for grain yield was also not related to cycle length or duration of grain filling but was due to increased number of grains per unit area.

## 5. Conclusions

Response of durum wheat varieties to preceding crop varied with prevailing weather conditions in the growing season. Overall, grain yield was 70% higher in the drier year. The durum wheat varieties in this study had similar reactions to the preceding crops. Wheat yields were influenced by previous crop. Preceding wheat exhibited the lowest yield, but this was not a factor of pests and disease incidence. Likewise, averaged over seasons and varieties, grain yield was increased by 48% when the preceding crop was alfalfa compared to wheat as preceding crop. This may be a favourable sequence for producers. This residual effect over years should be considered when formulating fertiliser requirements. Therefore, a better insight into the factors affecting the dynamics of immobilisation and remineralisation of fertiliser N is needed.

## Figures and Tables

**Table 1:** Accumulated growing degree days (GDD) and rainfall during the whole wheat cycle and main growth periods. Growth stages are defined following Zadok's scale [13].

Period	GDD (°C d)		Rainfall (mm)	
	2007-2008	2008-2009	2007-2008	2008-2009
GS00–GS10∗	174.6	139.6	85.2	169.2
GS10–GS15	405.8	381.5	191.6	206.4
GS15–GS30	270.5	233.6	38.6	161.2
GS30–GS60	751.4	793.3	103.2	141.6
GS60–GS90	1039.7	1075.6	140.6	69.2
Whole cycle	2641.9	2622.5	559.2	747.6

^*^GS00 seeding, GS10 emergence, GS15 5th leaf unfolded stage, GS30 pseudostem erection, GS60 anthesis, and GS90 physiological maturity.

**Table 2:** Effects of the preceding crop on preplant residual soil nitrate N (0–40 cm depth).

Preceding crop	Residual soil nitrate N (mg kg^−1^)	
	2007	2008
Alfalfa	12.3^a^	10.6^a^
Maize	7.9^b^	7.1^b^
Sunflower	6.5^b^	5.7^b^
Wheat	4.2^c^	3.3^c^

Within columns, numbers followed by the same letter are not significantly different at *P* ≤ 0.05.

**Table 3:** Grain yield, vegetative growth, and kernel number per spike. Year × preceding crop interaction.

Treatments	Preceding crop	Grain yield	Vegetative growth	Kernels per spike
Year		(g m^−2^)	(g m^−2^)	(*n* spike^−1^)
2007-2008	Alfalfa	573.2^a^	631.7^a^	34.6^a^
	Maize	462.2^b^	550.9^abc^	28.9^bc^
	Sunflower	497.3^b^	585.0^ab^	29.6^b^
	Wheat	300.7^c^	431.4^cd^	23.4^d^

2008-2009	Alfalfa	281.1^c^	364.8^d^	25.0^cd^
	Maize	326.2^c^	472.3^bcd^	24.5^d^
	Sunflower	294.3^c^	397.3^d^	24.8^d^
	Wheat	276.4^c^	401.7^cd^	26.9^bcd^

Within columns, numbers followed by the same letter are not significantly different at *P* ≤ 0.05.

**Table 4:** Mean values of spike number, mean kernel weight, spike fertility index, and spikelet number per spike as affected by year, preceding crop, and variety.

Treatments	Spike number	Mean kernel weight	Spike fertility index∗	Spikelet number
	n m^−2^	mg	n g^−1^	n spike^−1^
Year				
2007-2008	312.4^a^	52.7^a^	57.3^a^	17.8^a^
2008-2009	335.7^b^	36.2^b^	68.8^b^	17.2^a^
Preceding crop				
Alfalfa	318.7^a^	43.7^a^	70.7^a^	17.7^a^
Maize	328.1^a^	45.4^a^	59.6^b^	17.8^a^
Sunflower	325.5^a^	44.2^a^	59.0^b^	18.0^a^
Wheat	263.1^b^	44.5^a^	52.1^b^	16.4^b^
Variety				
Levante	324.3^a^	44.5^a^	60.6^a^	19.2^a^
Normanno	308.5^a^	44.1^a^	59.5^a^	17.1^b^
Saragolla	279.1^a^	43.1^a^	58.2^a^	18.1^ab^
Svevo	323.2^a^	46.0^a^	63.1^a^	15.6^c^

^*^Spike fertility index: grain number m^−2^/spike chaff dry weight m^−2^ at maturity.

Within treatments year, variety, and preceding crop, numbers followed by the same letter are not significantly different at *P* ≤ 0.05.

**Table 5:** Grain yield, vegetative plant part, and kernel number per spike. Year × variety interaction.

Treatments	Variety	Grain yield	Vegetative plant part	Kernels per spike
Year		(g m^−2^)	(g m^−2^)	(n spike^−1^)
2007-2008	Levante	570.0^a^	619.4^a^	32.9^a^
	Normanno	343.9^c^	457.1^bc^	23.7^cd^
	Saragolla	455.0^b^	501.0^b^	35.8^a^
	Svevo	464.5^b^	621.5^a^	26.2^bc^

2008-2009	Levante	307.1^cd^	445.4^bcd^	26.4^bc^
	Normanno	305.2^cd^	451.2^bcd^	25.2^bcd^
	Saragolla	302.7^cd^	375.6^cd^	28.0^b^
	Svevo	263.1^d^	363.8^d^	21.7^d^

Within columns, numbers followed by the same letter are not significantly different at *P* ≤ 0.05.

## References

[1] Tanaka D. L., Krupinsky J. M., Liebig M. A., Merrill S. D., Ries R. E., Hendrickson J. R., Johnson H. A., Hanson J. D. (2002). Dynamic cropping systems: an adaptable approach to crop production in the Great Plains. *Agronomy Journal*.

[2] Kirkegaard J., Christen O., Krupinsky J., Layzell D. (2008). Break crop benefits in temperate wheat production. *Field Crops Research*.

[3] Krupinsky J. M., Tanaka D. L., Merrill S. D., Liebig M. A., Hanson J. D. (2006). Crop sequence effects of 10 crops in the northern Great Plains. *Agricultural Systems*.

[4] Nix J., Hill P., Edwards A. (2006). *Farm Management Pocketbook*.

[5] Hargrove W. L., Touchton J. T., Johnson J. W. (1983). Previous crop influence on fertilizer nitrogen requirements for double-cropped wheat. *Agronomy Journal*.

[6] Smith S. J., Sharpley A. N. (1993). Nitrogen availability from surface applied and soil-incorporated crop residues. *Agronomy Journal*.

[7] Staggenborg S. A., Whitney D. A., Fjell D. L., Shroyer J. P. (2003). Seeding and nitrogen rates required to optimize winter wheat yields following grain sorghum and soybean. *Agronomy Journal*.

[8] Christen O., Sieling K., Hanus H. (1992). The effect of different preceding crops on the development, growth and yield of winter wheat. *European Journal of Agronomy*.

[9] Sieling K., Stahl C., Winkelmann C., Christen O. (2005). Growth and yield of winter wheat in the first 3 years of a monoculture under varying N fertilization in NW Germany. *European Journal of Agronomy*.

[10] Wu H., Pratley J., Lemerle D., Haig T. (2001). Allelopathy in wheat (*Triticum aestivum*). *Annals of Applied Biology*.

[11] Lithourgidis A. S., Damalas C. A., Gagianas A. A. (2006). Long-term yield patterns for continuous winter wheat cropping in northern Greece. *European Journal of Agronomy*.

[12] Procházková B., Hrubý J., Dovrtěl J., Dostál O. (2003). Effects of different organic amendment on winter wheat yields under long-term continuous cropping. *Plant, Soil and Environment*.

[13] Zadoks J. C., Chang T. T., Konzak C. F. (1974). A decimal code for the growth stages of cereals. *Weed Research*.

[14] Moonen C., Masoni A., Ercoli L., Mariotti M., Bonari E. (2001). Long-term changes in rainfall and temperature in Pisa, Italy. *Agricoltura Mediterranea*.

[15] Gee G. W., Bauder J. W., Klute A. (1986). Particle-size analysis. *Methods of Soil Analysis. Part 1. Physical and Mineralogical Methods*.

[16] McLean E. O., Page A. L., Miller R. H., Keeney D. R. (1982). Soil pH and lime requirement. *Methods of Soil Analysis Part 2: Chemical and Microbiological Properties, Agronomy Monograph*.

[17] Nelson D. W., Sommers L. E., Page A. L., Miller R. H., Keeney D. R. (1982). Total carbon, organic carbon and organic matter. *Methods of Soil Analysis, Part 2, Chemical and Microbiological Properties, Agronomy Monograph*.

[18] Bremner J. M., Mulvaney C. S., Page A. L., Miller R. H., Keeney D. R. (1982). Nitrogen-total. *Methods of Soil Analysis, Part 2, Chemical and Microbiological Properties*.

[19] Keeney D. R., Nelson D. W., Page A. L., Miller R. H., Keeney D. R. (1982). Nitrogen in organic forms. *Methods of Soil Analysis. Part 2. Chemical and Microbiological Properties*.

[20] Olsen S. R., Sommers L. E., Page A. L., Miller R. H., Keeney D. R. (1982). Phosphorus. *Methods of Soil Analysis, Part 2: Chemical and Microbiological Properties*.

[21] Thomas G. W., Page A. L., Miller R. H., Keeney D. R. (1982). Exchangeable cations. *Methods of Soil Analysis, Part 2, Chemical and Microbiological Properties, Agronomy Monograph*.

[22] McMaster G. S., Wilhelm W. W. (1997). Growing degree-days: one equation, two interpretations. *Agricultural and Forest Meteorology*.

[23] Porter J. R., Gawith M. (1999). Temperatures and the growth and development of wheat: a review. *European Journal of Agronomy*.

[24] Abbate P. E., Pontaroli A. C., Lázaro L., Gutheim F. (2013). A method of screening for spike fertility in wheat. *Journal of Agricultural Science*.

[25] Steel R. G. D., Torrie J. H., Dickey D. A. (1997). *Principles and Procedures of Statistics: A Biometrical Approach*.

[26] Karlen D. L., Varvel G. E., Bullock D. G., Cruse R. M. (1994). Crop rotations for the 21st century. *Advances in Agronomy*.

[27] Siqueira J. O., Nair M. G., Hammerschmidt R., Safir G. R. (1991). Significance of phenolic compounds in plant-soil-microbial systems. *Critical Review in Plant Sciences*.

[28] Bennett A. J., Bending G. D., Chandler D., Hilton S., Mills P. (2012). Meeting the demand for crop production: the challenge of yield decline in crops grown in short rotations. *Biological Reviews*.

[29] Gaiser T., Perkons U., Küpper P. M., Puschmann D. U., Peth S., Kautz T., Pfeifer J., Ewert F., Horn R., Köpke U. (2012). Evidence of improved water uptake from subsoil by spring wheat following lucerne in a temperate humid climate. *Field Crops Research*.

[30] Acreche M. M., Briceño-Félix G., Sánchez J. A. M., Slafer G. A. (2008). Physiological bases of genetic gains in Mediterranean bread wheat yield in Spain. *European Journal of Agronomy*.

[31] Slafer G. A., Andrade F. H. (1993). Physiological attributes related to the generation of grain yield in bread wheat cultivars released at different eras. *Field Crops Research*.

[32] Power J. F., Doran J. W., Wilhelm W. W. (1986). Uptake of nitrogen from soil, fertilizer, and crop residues by no- till corn and soybean.. *Soil Science Society of America Journal*.

[33] Carpenter-Boggs L., Pikul J. L., Vigil M. F., Riedell W. E. (2000). Soil nitrogen mineralization influenced by crop rotation and nitrogen fertilization. *Soil Science Society of America Journal*.

[34] Klem K., Váňová M., Hajšlová J., Lancová K., Sehnalová M. (2007). A neural network model for prediction of deoxynivalenol content in wheat grain based on weather data and preceding crop. *Plant, Soil and Environment*.

[35] Schaafsma A. W., Hooker D. C., Miller J. D. (2005). Progress and limitations with respect to pre-harvest forecasting of *Fusarium* toxins in grains. *Phytopathology*.

